# Effect of dural puncture epidural combined with programmed intermittent epidural bolus on labor analgesia in patients with gestational hypertension: a randomized controlled clinical trial

**DOI:** 10.3389/fmed.2025.1653301

**Published:** 2025-11-11

**Authors:** Binghui Zhang, Hongyang Zhang, Yuan Wu, Guofang Li, Shuxiang Liu, Kai Zhao

**Affiliations:** Department of Anesthesiology, The Fourth Hospital of Shijiazhuang, Shijiazhuang, China

**Keywords:** analgesia, epidural, anesthesia, spinal, obstetrical, infusions, hypertension, pregnancy-induced

## Abstract

**Background:**

Hypertensive disorders of pregnancy affect 5–10% of pregnancies and require the maintenance of hemodynamic stability while providing effective labor analgesia. This study compared the efficacy and safety of dural puncture epidural (DPE) block combined with programmed intermittent epidural bolus (PIEB) versus conventional epidural (EP) block in labor analgesia for patients with gestational hypertension (GH).

**Methods:**

Between January and March 2025, 98 primiparous women with GH and singleton pregnancies who requested neuraxial analgesia were randomized to receive either DPE–PIEB (Group D, *n* = 49) or EP–PIEB (Group E, *n* = 49). The primary outcome was time to effective analgesic onset (defined as Visual Analog Scale score≤30 mm). Secondary outcomes included hemodynamic stability, patient-controlled epidural analgesia (PCEA) use, incidence of breakthrough pain, maternal and infant outcomes, and adverse events.

**Results:**

Compared with EP–PIEB, patients receiving DPE–PIEB had a shorter onset of analgesia (6.05 ± 1.08 vs. 9.75 ± 1.3 min, *p* < 0.001), a longer time to first PCEA request (144.33 ± 17.18 vs. 116.58 ± 14.03 min, *p* < 0.001), fewer PCEA demands (2.78 ± 0.83 vs. 4.53 ± 1.26, *p* < 0.001), and had a lower incidence of breakthrough pain (9.1% vs. 25%, *p* < 0.05). The repeated measures ANOVA demonstrated that patients in Group D maintained lower and more consistent Mean arterial pressure (MAP) values throughout labor. MAP values were significantly lower at time points T1, T3, T4, and T5 in the DPE–PIEB group (*p* < 0.05), and maternal satisfaction scores were higher (9.39 ± 0.75 vs. 9.02 ± 0.76, *p* < 0.05). No significant between-group differences were found in neonatal outcomes (Apgar score, umbilical artery pH) or the incidence of adverse events between the two groups (*p* > 0.05).

**Conclusion:**

DPE–PIEB can significantly shorten the onset of labor analgesia in patients with GH, reduce hemodynamic fluctuations and breakthrough pain, and improve maternal satisfaction, without increasing maternal or neonatal risks. This combined technique provides a more optimized analgesic strategy and can be safely and effectively implemented in labor analgesia for patients with GH.

**Clinical trial registration:**

Identifier ChiCTR2400095084 (www.chictr.org.cn).

## Introduction

Hypertensive disorders of pregnancy, which affect 5–10% of pregnancies, are one of the leading causes of maternal and perinatal mortality (1, 2), and the associated hemodynamic instability poses a considerable challenge in the management of labor analgesia. Patients with gestational hypertension (GH) often exhibit small vessel spasms and vascular endothelial damage (3), which leads to increased sympathetic nerve activity and weakened autonomic regulation. These pathophysiological changes predispose patients to perinatal hemodynamic decompensation, subsequently compromising placental blood perfusion. Therefore, in patients with GH, labor analgesia must strike a careful balance—effectively relieving pain while minimizing hemodynamic fluctuations—to reduce the risk of eclampsia or placental perfusion insufficiency.

Although neuraxial analgesia is the gold standard for labor analgesia (4, 5), the traditional epidural (EP) block has the disadvantages of slow onset of action and a high incidence of analgesic insufficiency (6, 7), which leads to catecholamine surge and hemodynamic fluctuations, thus increasing perinatal risk. Studies have shown that the dural puncture epidural (DPE) block can rapidly achieve analgesia by puncturing the dura mater and allowing the local anesthetic to diffuse directly into the subarachnoid space. Compared with EP, DPE has been shown to reduce the time to onset of analgesia and improve the completeness of the block (8–10). However, when combined with traditional continuous epidural infusion (CEI), DPE may still result in breakthrough pain and local anesthetic accumulation. In recent years, programmed intermittent epidural bolus (PIEB) has been increasingly used in labor analgesia. Compared to CEI, PIEB delivers the anesthetic in pulsatile doses, which helps maintain more stable analgesia, reduces the incidence of breakthrough pain and motor block, and reduces the overall consumption of local anesthetic (11, 12). Preliminary evidence suggests that the combination of DPE and PIEB may enhance the quality of labor analgesia (13).

However, most of the existing studies are focused on healthy parturients. The pathophysiological states specific to patients with GH, including small vessel spasms and vascular endothelial damage, hyperexcitability of sympathetic nerves, and poor tolerance to hemodynamic fluctuations, may alter the diffusion and absorption of neuraxial anesthetics, as well as their cardiovascular effects. Therefore, although DPE and PIEB have demonstrated advantages in healthy parturients, their efficacy and safety in patients with GH remain unclear. Currently, there is a lack of high-quality evidence regarding the analgesic efficacy, maternal hemodynamic impact, and fetal safety of DPE-PIEB in patients with GH.

We hypothesized that the combination of DPE and PIEB may achieve a more effective balance of “effective analgesia and stable circulation” required for patients with GH through the dual mechanism advantages of “dural puncture hole promoting drug penetration” and “intermittent pulse pressure optimizing drug diffusion.” To test this hypothesis, we conducted this prospective randomized controlled trial to evaluate the efficacy, safety, and impact of this technique on maternal and neonatal outcomes. The ultimate objective is to provide evidence-informed, individualized analgesic protocols for this high-risk population.

## Methods

### Study design

This prospective, double-blind, randomized controlled trial was approved by the Medical Ethics Committee of the Fourth Hospital of Shijiazhuang (Approval No. 20240010), and registered with the Chinese Clinical Trial Registry on December 31, 2024 (Registration No. ChiCTR2400095084). The trial was conducted by the Declaration of Helsinki. All participants provided written informed consent before enrollment. We strictly adhered to the Consolidated Standards of Reporting Trials (CONSORT) guidelines throughout the study design and implementation. The first participant was enrolled on January 1, 2025, and the last participant was enrolled on March 25, 2025.

### Inclusion and exclusion criteria

A total of 98 full-term, primigravid women with hypertensive disorders (defined as systolic blood pressure≥140 mmHg and/or diastolic blood pressure≥90 mmHg) were enrolled for labor analgesia. All participants were aged between 22 and 40 years, had a BMI ≤ 35 kg/m^2^, and were classified as ASA physical status II. The exclusion criteria were known allergy to local anesthetics, use of analgesics within 4 h before labor analgesia, platelet count below 100 × 10^9^/L, or any contraindication to intrathecal anesthesia. The withdrawal criteria were the occurrence of serious adverse reactions, unintended dural puncture by the epidural needle, dural puncture by the spinal needle without cerebrospinal fluid (CSF) outflow, or patient withdrawal of consent.

### Randomization

Using computer-generated randomization (Stata Statistical Software, Release V.14, StataCorp), patients were consecutively randomized and assigned to either the DPE–PIEB group (Group D, *n* = 49) or the EP–PIEB group (Group E, *n* = 49). Allocation numbers were sealed in envelopes corresponding to the serial number of each study. These envelopes were opened by the anesthesiologist immediately before the administration of labor analgesia, thus, only the performing anesthesiologist was aware of the group assignment. Patients were not informed of their specific group allocation. Outcome assessments were performed by a research assistant who was blinded to group allocation and was not involved in the administration of anesthesia. In addition, the obstetrician and midwives in charge of the patients were also blinded to group assignments. The integrity of the envelopes was regularly monitored by the researchers, and access to the randomization table was prohibited until the end of the study.

### Labor analgesia protocol and monitoring procedures

When the cervix was dilated to 2–3 cm, patients who met the inclusion criteria were transferred to the labor analgesia unit. After establishing peripheral venous access, a compounded sodium chloride solution was infused at a rate of 10 mL·kg^−1^·h^−1^. Vital signs and fetal heart changes were continuously monitored. The patient was placed in the left lateral decubitus position, and the L_2–3_ epidural space was accurately localized under ultrasound guidance. A median approach was then employed, and the epidural space was confirmed using the loss-of-resistance-to-air method.

### Interventions

**Figure 1:**
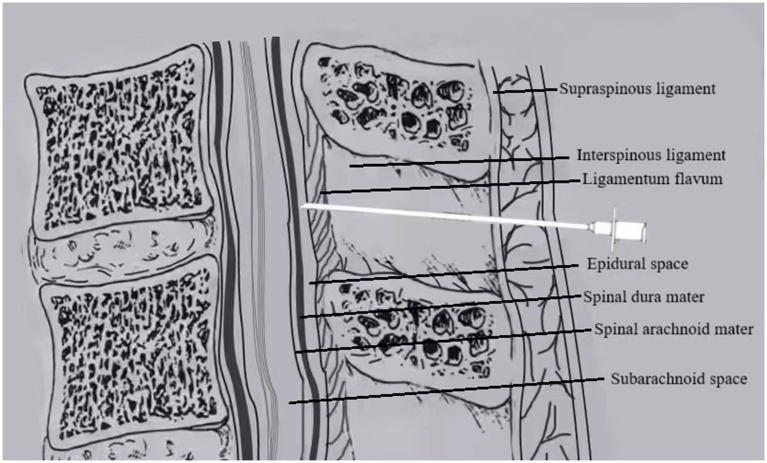
In EP, the epidural catheter is placed immediately after locating the epidural space with the Tuohy needle.

Group E (EP–PIEB): After successful epidural puncture, the epidural catheter was immediately inserted cephalad into the epidural space by 3–4 cm. The catheter was then secured after confirming the absence of CSF or blood on aspiration, as shown in Figure 1.

**Figure 2:**
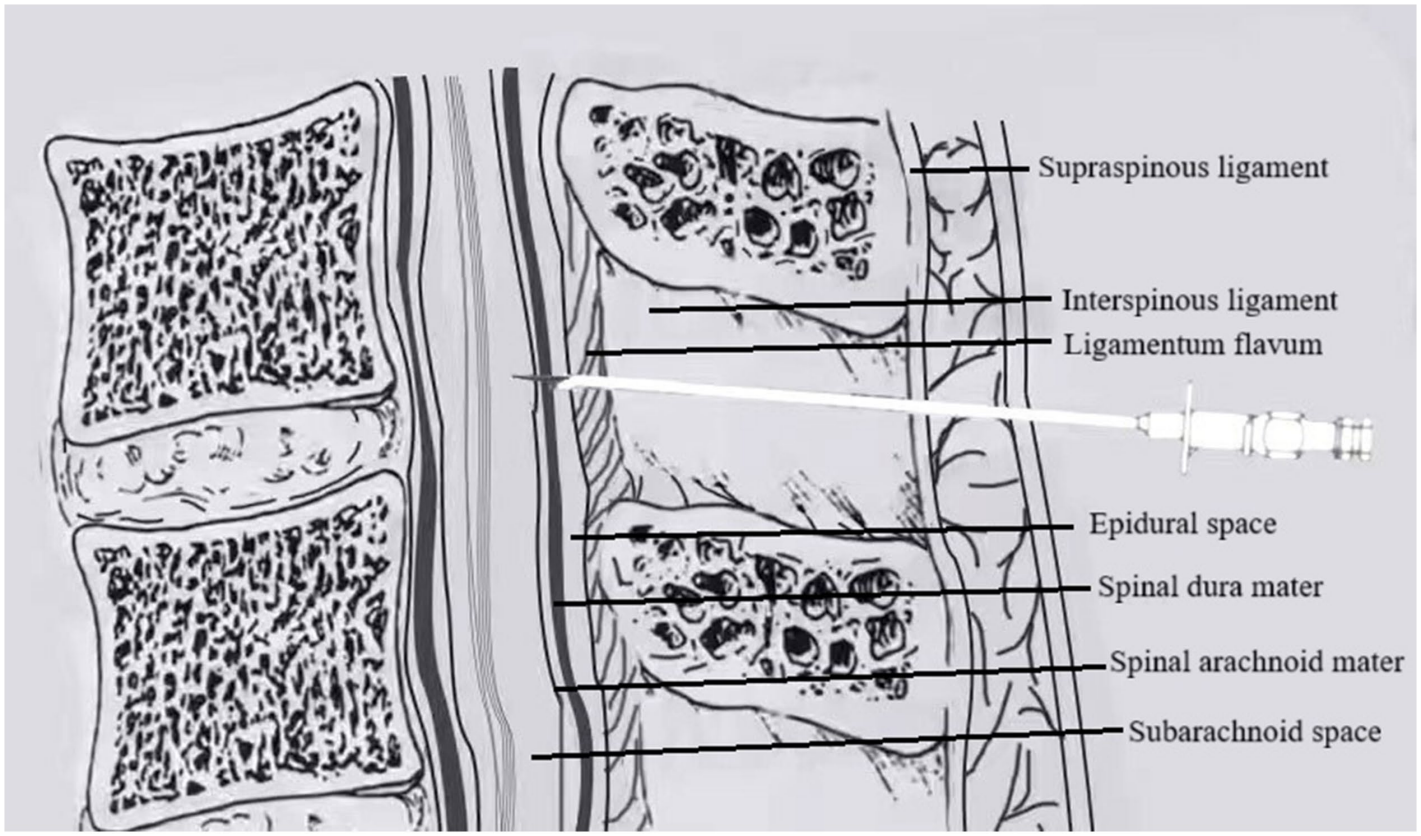
In DPE, a spinal needle (25G Whitacre) is first passed through the Tuohy needle to intentionally puncture the dura mater, creating a conduit, before the epidural catheter is threaded.

Group D (DPE–PIEB): After successful epidural puncture, the epidural catheter was temporarily withheld. A 25G Whitacre puncture needle was inserted through the Tuohy needle to puncture the dura mater. Once smooth CSF return was confirmed, the Whitacre needle was withdrawn. The epidural catheter was then advanced cephalad into the epidural cavity by 3–4 cm and securely fixed after ensuring that no CSF or blood was present upon aspiration, as shown in Figure 2.

### Analgesia administration protocol

After the procedure, patients were repositioned supine with left uterine displacement, and the head of the bed was elevated by 30°. A test dose of 3 mL of 1.5% lidocaine was administered, and after observing for 5 min to monitor for any adverse reactions, a loading dose consisting of 12 mL of 0.08% ropivacaine combined with 0.3 μg/mL of sufentanil was slowly injected over 2 min. The target sensory block level was at T10, assessed using the thermosensory method. A programmed epidural pulse infusion pump was connected 60 min after the first administration.

### Analgesia program parameters

The analgesic solution consisted of a 100-mL mixture of 0.08% ropivacaine compounded with 0.3 μg/mL of sufentanil. PIEB was delivered at a pulse dose of 10 mL every 60 min (14). Patient-controlled epidural analgesia (PCEA) was set to allow a 5-mL bolus with a 20-min lockout interval.

### Pain assessment and management

Patients were asked to rate their pain during intrathecal labor analgesia on a visual analog scale (VAS), where a score of 0 indicated no pain and 100 mm indicated unbearable pain. The scores were recorded at the following time points: before analgesia; at 10 min, 30 min, 60 min, and 2 h after analgesia; and at full cervical dilatation. Breakthrough pain, defined as a VAS score >40 mm after at least one pulse dose of PCEA and a patient request for additional analgesia, was managed with a 5-mL epidural bolus of 0.1% ropivacaine.

### Hemodynamic management protocol

Hemodynamic abnormalities, excluding cases where systolic blood pressure exceeded 20% of the baseline value, were initially managed with positional adjustments and fluid resuscitation. If these measures failed to provide relief, pharmacological interventions were administered as follows:

- Hypotension (systolic blood pressure <20% below baseline): intravenous (IV) ephedrine 5 mg per dose.
- Bradycardia (heart rate <50 bpm): IV atropine 0.5 mg per dose.
- Bradycardia (heart rate >120 bpm): IV esmolol 0.5 mg/kg.
- Hypertension (systolic blood pressure >20% of baseline value): IV urapidil 12.5 mg per dose.

### Outcomes

The primary outcome of the study was the time to effective analgesia onset after implementation of labor analgesia, defined as the time from the initiation of analgesic administration to the point when the patient reported a VAS score≤30 mm during two consecutive uterine contractions.

Secondary outcomes included:

1. Hemodynamic and pain assessments: Mean arterial pressure (MAP) and VAS scores were recorded at baseline (T0), 10 min (T1), 30 min (T2), 60 min (T3), 2 h (T4), and at full cervical dilatation (T5).
2. PCEA parameters: These included the time to first patient-initiated PCEA dose, the number of effective PCEA demands, and the incidence of breakthrough pain.
3. Motor blockade assessment: The degree of motor blockade was assessed using the modified Bromage scale (Grade 0: no motor block; Grade 1: inability to raise the extended leg; Grade 2: inability to flex the knee; and Grade 3: inability to flex the ankle). Assessments were performed at 20 min and 1 h after drug administration, followed by hourly evaluations, with all measurements conducted by assessors blinded to group allocation and not involved in anesthesia administration.
4. Labor and neonatal outcomes: These included the duration of the first and second stages of labor, mode of delivery (natural vaginal delivery, instrumental delivery, or cesarean section), neonatal Apgar scores at 1 and 5 min after birth, and the results of umbilical artery blood gas analysis at the delivery of the fetus.
5. Fetal heart rate (FHR) was continuously monitored via cardiotocography (CTG) from 30 min pre-analgesia until delivery. FHR abnormalities were defined as: (a) Late decelerations: Gradual, symmetrical decrease in FHR occurring near the peak of a uterine contraction, with≥30 s from the onset of the deceleration to its nadir; (b) Variable decelerations: Abrupt decrease in FHR following the onset of a uterine contraction, characterized by≥30 s from onset to nadir, a decrease of≥15 bpm from the baseline FHR, and a duration between 15 s and 2 min; (c) Prolonged decelerations: Decrease in baseline FHR of≥15 bpm lasting 2–10 min; (d) Tachycardia (>160 bpm) or bradycardia (<110 bpm) lasting >10 min.
6. Other adverse events: Documented events included nausea and vomiting, dramatic fluctuations in blood pressure (systolic blood pressure fluctuations >20%), bradycardia (HR < 50 bpm), tachycardia (HR > 120 bpm), pruritus, post-puncture headache, eclampsia, and the use of antihypertensive drugs.
7. Patient satisfaction: Assessed 24 h postpartum using a numerical rating scale from 0 to 10, where 0 indicated complete dissatisfaction and 10 indicated complete satisfaction.

### Sample size prediction

Based on a previous study by Wilson et al. (8), which reported that 50% of healthy parturients receiving an EP achieved a VAS score≤10 mm at 10 min compared to 80% of those receiving a DPE, we hypothesized that there would be a significant difference in the analgesia onset time between the DPE–PIEB and EP–PIEB techniques in patients with GH. Using a two-sided *α*-level of 2.5% and a power of 80%, the required sample size was calculated to be 44 patients in each group. Taking into account a patient dropout rate of 10%, we planned to enroll 98 participants (49 in Group D and 49 in Group E) in the clinical trial.

### Statistical analysis

Data were analyzed using SPSS 26.0 (IBM Corp., Armonk, NY, USA) and GraphPad Prism 9.0 (GraphPad Software, San Diego, CA, USA). Continuous variables were tested for normality with the Shapiro–Wilk test. Normally distributed data are presented as mean ± standard deviation and were compared between groups using independent samples *t*-tests (with Levene’s test for equality of variances). The Mann–Whitney *U*-tests were used if they did not conform to a normal distribution. To specifically assess hemodynamic stability, serial measurements of MAP across time points (T0–T5) were analyzed using repeated measures ANOVA. This model assessed the main effects of Group (DPE-PIEB vs. EP-PIEB) and Time, as well as the critical Group × Time interaction. A significant interaction was followed with Benjamini–Hochberg false discovery rate correction method. Categorical variables, including dichotomous, unordered categorical, and ordered categorical data, were analyzed using the Pearson *χ*^2^ test. Kaplan–Meier analysis was used to compare the time to onset of analgesia between groups, with between-group differences assessed using the log-rank test (Mantel–Cox method). The proportional risk hypothesis was tested using log–log survival plots. All statistical tests were two-sided, with *p* < 0.05 considered statistically significant. Statistical graphs were generated using GraphPad Prism 9.0 in accordance with journal data visualization specifications. Multiple comparisons were adjusted using the Benjamini–Hochberg false discovery rate correction method.

## Results

**Figure 3:**
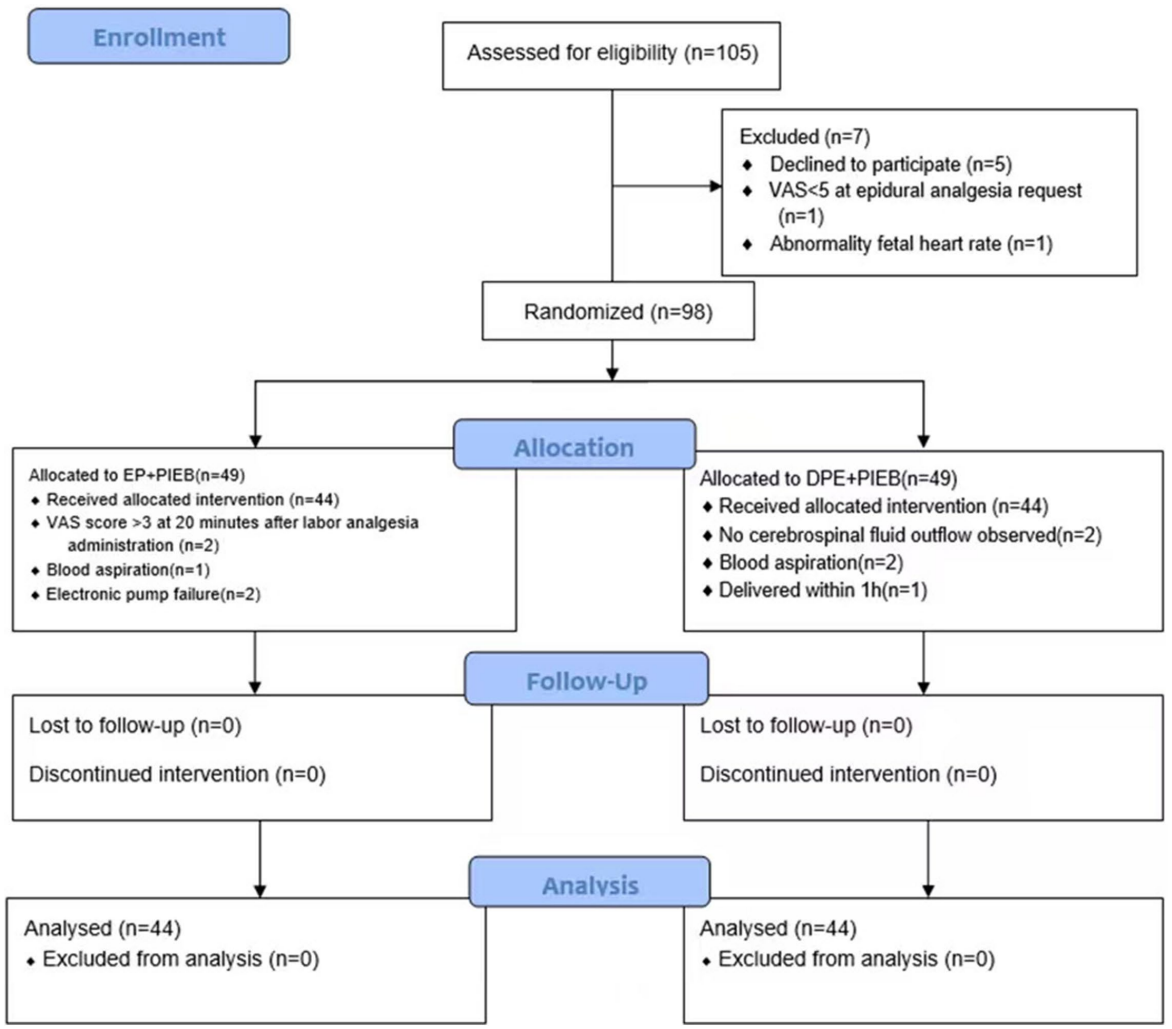
The CONSORT flow diagram. Exclusion, randomization, and eligibility for analysis. DPE, dural puncture epidural; EP, epidural; PIEB, programmed intermittent epidural bolus.

A total of 105 patients were screened for eligibility, of whom 98 met the inclusion criteria and were subsequently randomized. The flowchart of participant selection and enrollment was shown in Figure 3.

Table 1 shows the demographic characteristics and baseline parameters of the patients in the two groups.

**Table 1:** Demographic characteristics and baseline parameters in Groups D and E.

Characteristic	Group D (*n* = 44)	Group E (*n* = 44)	*p* value
Gestational age (weeks)	39.15 ± 0.99	39.18 ± 0.97	0.89
Height (cm)	165.09 ± 4.68	163.95 ± 5.15	0.28
Weight (kg)	79.83 ± 10.75	82.53 ± 9.81	0.22
BMI (kg/m^2^)	29.26 ± 3.48	30.71 ± 3.39	0.05
Age (years)	27.86 ± 3.14	28.45 ± 3.81	0.43
Baseline fetal heart rate (bpm)	140.34 ± 2.20	140.11 ± 2.30	0.64
Cervical dilation at analgesia initiation (cm)	2.91 ± 0.29	2.88 ± 0.33	0.61
VAS pain score at analgesia initiation (mm)	80.02 ± 5.42	80.57 ± 5.60	0.64
Baseline SBP (mmHg)	142.41 ± 4.01	142.11 ± 4.84	0.76
Baseline DBP (mmHg)	92.00 ± 3.89	91.32 ± 4.61	0.46
Baseline MAP (mmHg)	108.80 ± 2.59	108.27 ± 2.84	0.37

Statistical tests: *t*-test for continuous variables.

BMI, body mass index; VAS, visual analog scale; SBP, systolic blood pressure; DBP, diastolic blood pressure; MAP, mean arterial pressure; bpm, beats per minute.

### Primary outcome

**Figure 4:**
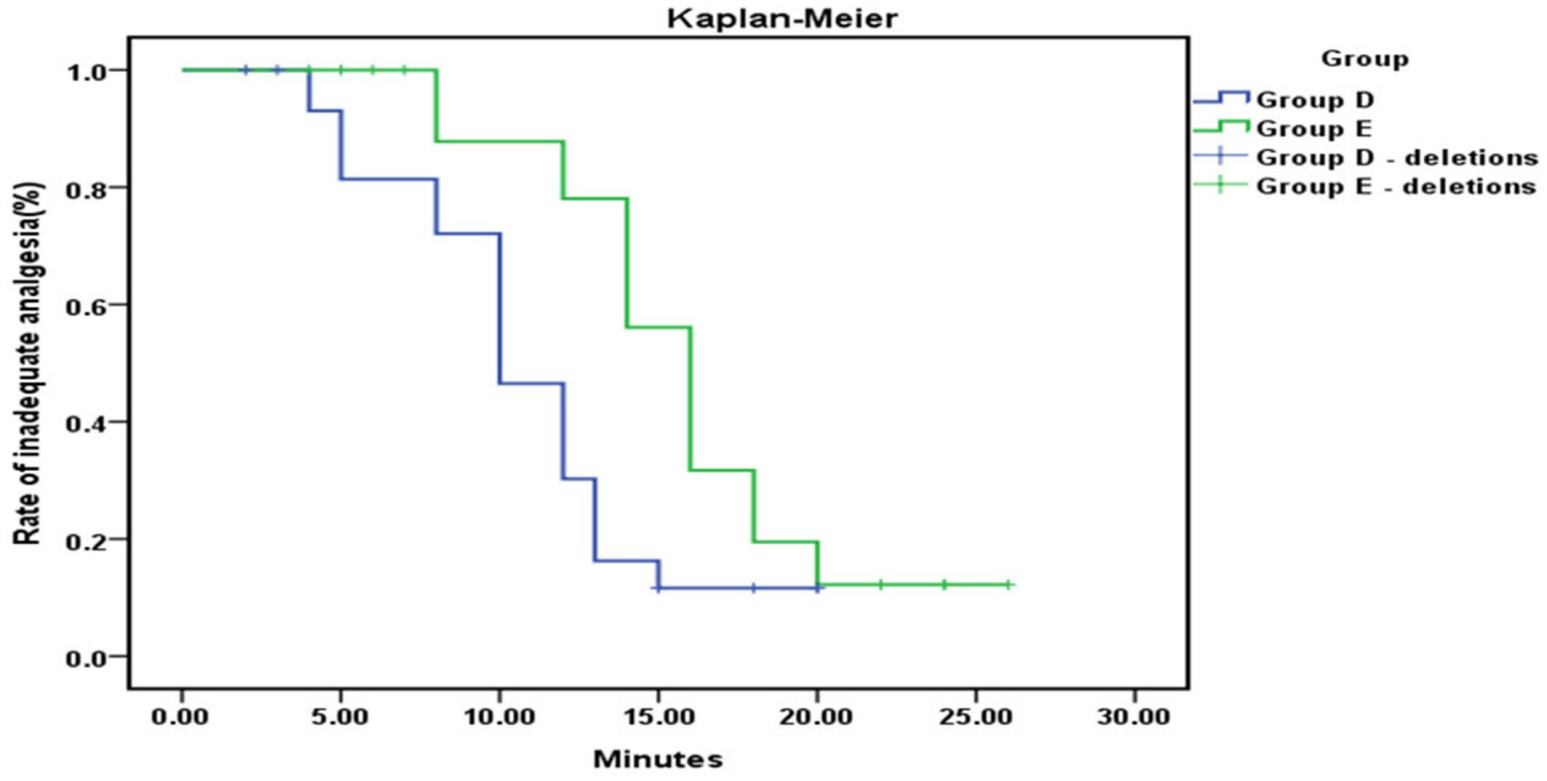
Kaplan–Meier analysis showed a significantly shorter time to effective analgesia from the loading dose in Group D compared to Group E (*p* < 0.001).

The time to onset of analgesia was significantly shorter in Group D compared to the control Group E (6.05 ± 1.08 vs. 9.75 ± 1.30 min, *p* < 0.001), as shown in Figure 4.

### Secondary outcomes

**Figure 5:**
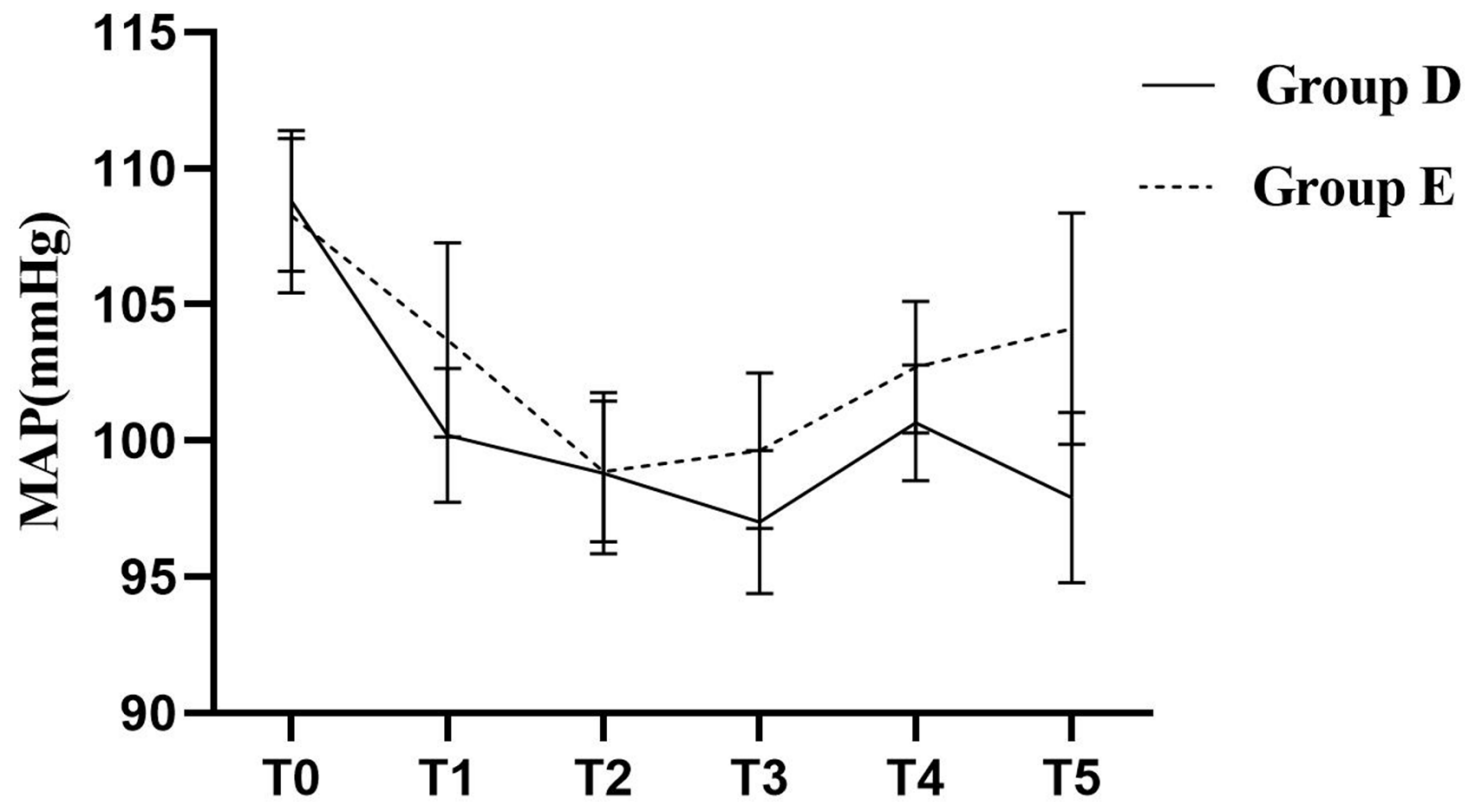
Trajectories of Mean Arterial Pressure (MAP) during labor in the two groups. A repeated-measures ANOVA revealed a significant Time × Group interaction effect (*p* < 0.05), indicating that the pattern of MAP change differed significantly between the two groups. The Group D demonstrated a lower and more stable hemodynamic profile.

The pattern of MAP change over time was significantly different between the two groups as assessed by repeated measures ANOVA (group-by-time interaction, *p* < 0.05). No instances of systolic blood pressure exceeding 20% of the baseline were observed in either of the groups. While MAP decreased to some extent in both groups after the implementation of labor analgesia, *post-hoc* analysis revealed that patients in Group E had significantly higher MAP values at time points T1, T3, T4, and T5 compared to Group D, and the difference was statistically significant (*p* < 0.05; Table 2). The hemodynamic profiles of both groups across all time points are visually represented in Figure 5. Specifically, MAP was 100.20 ± 2.47 mmHg in Group D and 103.70 ± 3.56 mmHg in Group E at the T1 time point; this trend persisted at the T3, T4, and T5 time points: T3 (97.00 ± 2.64 vs. 99.64 ± 2.85 mmHg); T4 (100.66 ± 2.13 vs. 102.70 ± 2.41 mmHg); T5 (97.90 ± 3.13 vs. 104.12 ± 4.25 mmHg).

**Table 2:** MAP values at each time point (T0–T5) for both groups.

Time point	Group D (*n* = 44)	Group E (*n* = 44)	*p* value
T0 (Baseline)	108.80 ± 2.59	108.27 ± 2.84	0.370
T1 (10 min)	100.20 ± 2.47	103.70 ± 3.56	<0.001
T2 (30 min)	98.80 ± 2.96	98.86 ± 2.59	0.909
T3 (60 min)	97.00 ± 2.64	99.64 ± 2.85	<0.001
T4 (2 h)	100.66 ± 2.13	102.70 ± 2.41	<0.001
T5 (full cervical dilatation)	97.90 ± 3.13	104.12 ± 4.25	<0.001

Statistical tests: a repeated measures ANOVA revealed a significant Group × Time interaction effect on MAP (*p* < 0.001).

**Figure 6:**
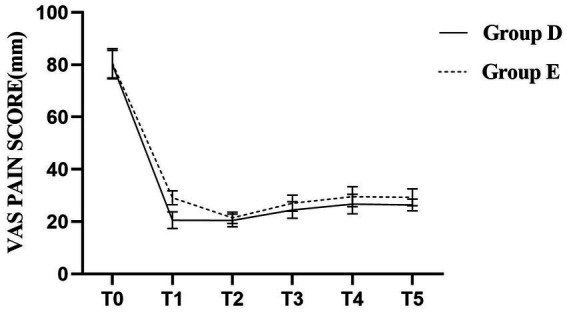
Parturients in Group D exhibited significantly lower VAS scores at T1, T3, T4, and T5 compared to Group E (*p* < 0.05).

The VAS scores for both groups at all predefined time points are summarized in Table 3. Compared to Group E, patients in Group D had significantly lower VAS scores at time points T1, T3, T4, and T5 (*p* < 0.05), indicating more effective pain control. No statistically significant differences were observed at the other time points (*p* > 0.05), as shown in Figure 6.

**Table 3:** VAS scores at each time point (T0–T5) for both groups.

Time Point	Group D (*n* = 44)	Group E (*n* = 44)	*p* value
T0 (Baseline)	80.02 ± 5.42	80.57 ± 5.60	0.644
T1 (10 min)	20.57 ± 3.17	29.16 ± 2.68	<0.001
T2 (30 min)	20.45 ± 2.39	21.48 ± 2.18	0.039
T3 (60 min)	24.43 ± 3.16	27.00 ± 3.13	<0.001
T4 (2 h)	26.68 ± 3.81	29.50 ± 3.91	<0.001
T5 (full cervical dilatation)	26.37 ± 2.28	29.26 ± 3.31	<0.001

Statistical tests: *t*-test for continuous variables.

Patients in Group D had a significantly longer time to first PCEA activation, fewer effective PCEA demands, and a lower incidence of breakthrough pain compared to Group E (*p* < 0.05). No statistically significant differences were observed between the groups in terms of Bromage scores, duration of the first and second stages of labor, mode of delivery, or umbilical artery blood gas pH (*p* > 0.05). Additionally, patients in Group D reported significantly higher satisfaction scores regarding the effect of analgesia on the first day after delivery (*p* < 0.05; Table 4). The Apgar scores at 1 and 5 min were 10 in both groups.

**Table 4:** Comparison of analgesia and delivery outcomes between Group D and E.

Outcome measure	Group D (*n* = 44)	Group E (*n* = 44)	*p* value
Onset time of analgesia (min)	6.05 ± 1.08	9.75 ± 1.30	<0.001
Time to first PCEA (min)	144.33 ± 17.18	116.58 ± 14.03	<0.001
Effective PCEA demands (n)	2.78 ± 0.83	4.53 ± 1.26	<0.001
Breakthrough pain (*n*, %)			0.047
No	40 (90.91%)	33 (75.00%)	
Yes	4 (9.09%)	11 (25.00%)	
Modified Bromage score	0.07 ± 0.25	0.07 ± 0.25	1.000
Delivery mode (*n*, %)			1.000
Spontaneous vaginal delivery	40 (90.91%)	40 (90.91%)	
Forceps-assisted delivery	1 (2.27%)	1 (2.27%)	
Cesarean section	3 (6.82%)	3 (6.82%)	
Duration of first labor stage (min)	466.43 ± 183.03	466.82 ± 157.06	0.992
Duration of second labor stage (min)	64.98 ± 42.83	60.02 ± 39.92	0.576
Umbilical artery pH	7.30 ± 0.07	7.32 ± 0.06	0.256
Patient satisfaction score	9.39 ± 0.75	9.02 ± 0.76	0.027

Statistical tests: *t*-test for continuous variables; *χ*^2^ test for categorical data.

PCEA, patient-controlled epidural analgesia; min, minutes; *n*, number.

There was no significant difference in the occurrence of adverse events between the two groups (*p* > 0.05). One patient in Group E experienced transient hypotension, which was effectively managed with positional adjustment and rehydration. No significant differences in FHR abnormalities were observed between groups (*p* > 0.05). All cases resolved spontaneously or with positional adjustment, with no emergent deliveries required. No cases of headache or eclampsia after epidural puncture were reported in either group (Table 5).

**Table 5:** Comparison of adverse events between groups D and E.

Adverse event	Group D (*n* = 44)	Group E (*n* = 44)	*p* value
Nausea/Vomiting	1 (2.27%)	2 (4.55%)	0.56
Pruritus	2 (4.55%)	2 (4.55%)	1.00
Abnormal FHR	2 (4.55%)	3 (6.82%)	0.65
Fever	2 (4.55%)	2 (4.55%)	1.00
Post-dural headache	0 (0%)	0 (0%)	1.00
Hypotension	0 (0%)	1 (2.27%)	0.24
Eclampsia	0 (0%)	0 (0%)	1.00

Statistical tests: categorical variables were compared using the *χ*^2^ test.

Data are presented as *n* (%).

## Discussion

The results of this study showed that the application of the combined DPE–PIEB technique significantly shortened the onset time of labor analgesia and provided more stable hemodynamics in patients with GH. Additionally, patients in the DPE–PIEB group experienced a longer time to first PCEA activation, fewer effective PCEA demands, and a lower incidence of breakthrough pain, leading to significantly higher satisfaction scores. There were no statistically significant differences between the two groups in terms of labor and neonatal outcomes or the overall occurrence of adverse events.

As introduced, patients with GH are characterized by small vessel spasms, vascular endothelial damage, and heightened sympathetic tone, making them particularly vulnerable to hemodynamic decompensation. The faster onset of profound analgesia observed in Group D likely contributes to a more rapid and effective suppression of labor pain-induced catecholamine release. This effect, combined with the more stable MAP profile, suggests that the DPE-PIEB technique not only provides superior pain relief but also maintains hemodynamic stability in patients with GH. This approach may help protect placental perfusion and reduce the risk of eclampsia, thereby fulfilling the critical balance of ‘effective analgesia and stable circulation’ required for this high-risk population.

The results demonstrated a significantly shorter onset time of analgesia in Group D compared to Group E. This finding aligns with previous research by Song et al., who reported that DPE significantly reduced the onset time of analgesia compared to EP (13). Lin et al. demonstrated that the DPE-PIEB combination (with a 25G spinal needle) for labor analgesia significantly accelerated the onset and enhanced the overall quality of analgesia (15). In addition, a meta-analysis that included a total of 1,099 parturients across 10 studies showed that DPE was associated with significantly lower VAS scores at 10 and 20 min after labor analgesia initiation compared to EP (16), which is consistent with the results of this study. These results suggest that the DPE can improve the quality of labor analgesia. The proposed mechanism underlying this improvement is that a dural puncture creates a small hole through which the local anesthetic can flow into the subarachnoid space, facilitated by a pressure gradient. This allows for a more rapid and effective onset of analgesia (17). Bernards et al., in an extracorporeal dural puncture epidural study in monkeys, observed that the flow of local anesthetic into the subarachnoid space was strongly correlated with the size of the puncture hole, the type of drug, and the distance between the puncture hole and the tip of the epidural catheter (18). Similarly, Cappiello et al. observed that analgesia may be more effective using a 25G Whitacre needle and a high-volume, low-concentration local anesthetic solution (9). Based on this evidence, a 25G Whitacre needle was chosen to puncture the dura mater in this study. The flow of anesthetic through the puncture hole is affected not only by the size of the puncture needle but also by the diffusion rate and penetration ability of the anesthetic drug in the epidural space. These factors are particularly critical in patients with GH, where the goal is to achieve adequate analgesia while avoiding minimizing hemodynamic fluctuations. Therefore, a combination of ropivacaine and sufentanil was chosen. Ropivacaine, known for its ability to separate motor and sensory blockade at low concentrations, is widely used in labor analgesia owing to its long duration of action and low cardiotoxicity (19). Sufentanil, a potent opioid receptor agonist, provides a synergistic effect when combined with ropivacaine, improving both the speed of onset and the duration of analgesia (20).

Patients with GH are prone to hemodynamic fluctuations due to weakened autonomic regulation, which can affect placental perfusion. In this study, MAP decreased to a certain extent in both groups after labor analgesia. However, the significant Group × Time interaction in MAP confirmed that the two groups followed distinct trajectories. Specifically, Group D experienced a more favorable hemodynamic course, as evidenced by lower MAP values at critical time points and a more stable overall profile, suggesting that this technique helps stabilize hemodynamics more effectively in patients with GH. Additionally, VAS scores were consistently lower in Group D at 10 min, 60 min, 2 h, and at full cervical dilatation, indicating superior analgesic efficacy throughout labor. Studies have shown that intrathecal labor analgesia can effectively reduce the release of catecholamines and decrease the pain-induced increases in cardiac output and blood pressure (21). The DPE technique facilitates the diffusion of local anesthetics through the dural puncture site. The more gradual and stable reduction in MAP observed with the DPE–PIEB technique, compared to EP–PIEB, may reflect a more controlled and progressive sympathetic blockade, thereby mitigating hemodynamic fluctuations. Furthermore, the intermittent bolus administration in PIEB enhances drug distribution within the epidural space, which minimizes local anesthetic accumulation, reduces the risk of motor blockade, and maintains effective sensory analgesia. The PIEB technique, first described by Wong et al. in 2006, is gaining popularity in labor analgesia. Unlike continuous epidural infusion, PIEB delivers controlled boluses of local anesthetic into the epidural space at programmed intervals rather than a continuous flow. Hussain et al. conducted a meta-analysis of 27 studies and concluded that intermittent epidural injections improve the quality of labor analgesia during the first 4 h of administration (22). In the present study, the combination of DPE and PIEB was associated with a lower incidence of breakthrough pain and a longer duration before the first PCEA demands compared to the conventional epidural block. These findings are consistent with those of Song et al., who concluded that DPE combined with PIEB not only reduces the incidence of breakthrough pain but also offers a high safety profile for labor analgesia (13). In recent years, studies have also shown that the PIEB infusion mode can reduce the frequency of PCEA use, thus reducing the total amount of local anesthetic required (23), which is consistent with the results of the present study. These results suggest that the combination of DPE and PIEB not only reduces the incidence of breakthrough pain but may also reduce the release of catecholamines in patients with GH. The pulsatile infusion pattern in PIEB can increase the speed of epidural drug delivery by increasing the pulse infusion pressure, which can lead to the wide diffusion of the local anesthetic and reduce its dosage, reduce the incidence of hypotension, and improve the analgesic effect (24, 25). Furthermore, because DPE involves the intentional puncture of the dura mater to confirm the CSF reflux, it allows for more accurate identification of the midline epidural space. This contributes to more precise catheter placement and may reduce the incidence of incomplete or failed blocks.

The present study showed that labor and neonatal outcomes and adverse events did not show statistically significant differences between the two groups of patients. These findings are consistent with those of Bullingham et al., who reported that PIEB administration does not affect the mode of delivery (26). Similarly, Song et al. concluded that the use of DPE for intrathecal labor analgesia does not increase maternal or neonatal side effects (13), which is consistent with the results of this study. These results suggest that the DPE–PIEB approach does not prolong labor or elevate the risk of adverse maternal or neonatal outcomes. However, our study also found that patients in the DPE–PIEB group had significantly higher satisfaction scores with the analgesic experience.

This study has several limitations. First, it was a single-center trial with a relatively small sample size, which may introduce potential selection bias. Although standardized protocols and rigorous blinding were implemented to minimize bias, future multi-center studies are warranted to validate our findings. Second, our study did not include long-term follow-up of maternal or neonatal outcomes, such as the resolution of postpartum hypertension or infant neurodevelopment. Future research should incorporate extended assessments to comprehensively evaluate the long-term safety of the DPE–PIEB technique. Third, variations in drug doses, concentrations, pulse intervals, and injection speeds of the PIEB pump may influence the results, and future studies should explore optimized infusion parameters. Furthermore, this study did not account for non-physiological factors such as maternal anxiety, pre-existing expectations for labor analgesia, and individual variability in pain thresholds. These psychosocial elements may have significantly influenced patient-reported outcomes, including VAS pain scores and satisfaction ratings, potentially introducing subjective bias that could not be controlled by our standardized analgesic protocol. To address these limitations, future research will involve larger, multicenter trials encompassing diverse populations, including pregnant patients with various subtypes of hypertensive disorders (such as preeclampsia and chronic hypertension) across different ethnicities and regions. These studies will aim to further evaluate the efficacy of DPE–PIEB in managing different hypertensive disorders during pregnancy, while optimizing analgesic protocols to achieve more precise, individualized pain management strategies.

## Conclusion

The combination of DPE and PIEB can optimize the effect of labor analgesia in patients with GH. This approach maintains perinatal hemodynamic stability and does not adversely affect maternal or neonatal outcomes. Therefore, DPE–PIEB can be considered a safe and effective strategy for labor analgesia in patients with GH.

## Data Availability

The raw data supporting the conclusions of this article will be made available by the authors, without undue reservation.
